# Virome analysis of Desmodus rotundus tissue samples from the Amazon region

**DOI:** 10.1186/s12864-023-09950-w

**Published:** 2024-01-04

**Authors:** Nádia K. Albuquerque, Sandro P. Silva, Carine F. Aragão, Tânia Cristina A. S. Cunha, Francisco A. S. Paiva, Taciana F. S. B. Coelho, Ana Cecília R. Cruz

**Affiliations:** 1 Institute of Biologic Science, Federal University of Pará, Augusto Corrêa Road, Belém, 66075-750 Pará Brazil; 2 Arbovirology and Hemorragic Fever Department, Evandro Chagas Institute, BR-316 Highway, Ananindeua, 67030-000 Pará Brazil

**Keywords:** Metagenomic, *Desmodus rotundus*, Bats, Viruses

## Abstract

**Background:**

Bats are renowned for harboring a high viral diversity, their characteristics contribute to emerging infectious diseases. However, environmental and anthropic factors also play a significant role in the emergence of zoonotic viruses. Metagenomic is an important tool for investigating the virome of bats and discovering new viruses.

**Results:**

Twenty-four families of virus were detected in lung samples by sequencing and bioinfomatic analysis, the largest amount of reads was focused on the *Retroviridae* and contigs assembled to *Desmodus rotundus endogenous retrovirus*, which was feasible to acquire complete sequences. The reads were also abundant for phages.

**Conclusion:**

This lung virome of *D. rotundus* contributes valuable information regarding the viral diversity found in bats, which is useful for understanding the drivers of viral cycles and their ecology in this species. The identification and taxonomic categorization of viruses hosted by bats carry epidemiological significance due to the potential for viral adaptation to other animals and humans, which can have severe repercussions for public health. Furthermore, the characterization of endogenized viruses helps to understanding the host genome and the evolution of the species.

**Supplementary Information:**

The online version contains supplementary material available at 10.1186/s12864-023-09950-w.

## Introduction

Chiropterans represent the second most diverse mammalian order in the world after rodents [1]. They are renowned for harboring a high viral diversity among mammals, their migratory ability, longevity and unique immunology contribute to emerging infectious diseases. However, environmental and anthropic factors also play a significant role in the emergence of zoonotic viruses [2]. Deforestation, environmental disasters, climatic changes, urbanization and human economic and cultural activities increase the contact with the viral diversity of bats and the occurrence of many outbreaks caused by families such as *Paramyxoviridae, Filoviridae, Rhabdoviridae*, and *Coronaviridae*, including the recent caused by SARS-CoV-2 in 2020 [3, 4].

Amazon forest has a greater richness of bat species, Brazil exhibits a substantial portion of the recorded, with a total of 181 species distributed across 68 genera and 9 families, according to the latest list provided by the Brazilian Society for Chiropteran Studies [5], mainly related to *Phyllostomidae, Vespertilionidae* and *Molossidae*, they are important to polinization, seed dispertion and insect predation [6, 7]. The state of Pará, has the most records of species in North of Brazil, *Desmodus rotundus* is commonly used in research due to their role as reservoirs of *Lyssavirus rabies* in Amazon region [8, 9]. Environmental disturbances have modified the interactions and virome composition of this specie, hematophagous bats have a feeding dynamic that facilitates the transmission of viruses to wild or domestic animals, as well as directly to humans, particularly those residing in communities around rivers and forests. Extractive activities, agriculture and agribusiness in Amazon cause habitat and bat colony destruction, leaving local populations more susceptible to spillover [4].

The decreasing costs of Next-Generation Sequencing (NGS) combined with the need for research on emerging pathogens has resulted in a focused effort to discover viruses in bats. This has led to the identification of entire clades of viruses, with metagenomics providing a broader perspective and allowing for the detection of new microorganisms beyond the main disease-causing agents [10]. In recent years, numerous viruses have been discovered in bats using NGS, with around 30% of bat-related viral sequences in GenBank obtained through metagenomics, primarily consisting of RNA viruses. RNA viruses are prone to mutation, which increases their pathogenicity and severity of the resulting effects. Urine, saliva, and feces samples are commonly used in studies as they have a lower impact on bat populations and represent possible transmission routes. However, relying solely on these samples may lead to the failure to detect viruses associated with specific tissues or environmental interference in the results [11].

Monitoring the virome in animals that are important reservoirs assists in identifying viruses with pathogenic potential. Knowing about viruses hosted by bats in the Amazon region is a crucial measure for virological surveillance and may serve as a preliminary step in detecting potential zoonotic viruses in circulation. Studies that utilize NGS for this purpose have the capacity to provide valuable insights that can help mitigate the impact of future epidemics or pandemics [9, 11].

Currently, studies involving samples from bats inhabiting Amazonian regions have already identified numerous viral families related to different taxa, such as insect, plant, protozoan, vertebrate viruses, and a substantial quantity of bacteriophages, through metagenomic analyses [12, 13]. Families that harbor zoonotic viruses are always highlighted, however, bats also play a role as hosts of retroviruses. Thus, detecting Endogenous Retroviruses (ERVs) can contribute to understanding evolutionary aspects of species, gaining insights into the potential development of cancer, neurological diseases, and autoimmune conditions, as observed in other mammals ERVs [14, 15].

The purpose of this investigation was to detect RNA viruses in the lung samples from brazilian *D. rotundus* bats. Moreover, to perform a comparative analysis of the identified viruses with those present in genomic sequence databases from humans and/or other animals and taxonomically classify them using phylogenetic analysis.

## Results

### Sequencing and bioinformatic analyses

**Fig. 1:**
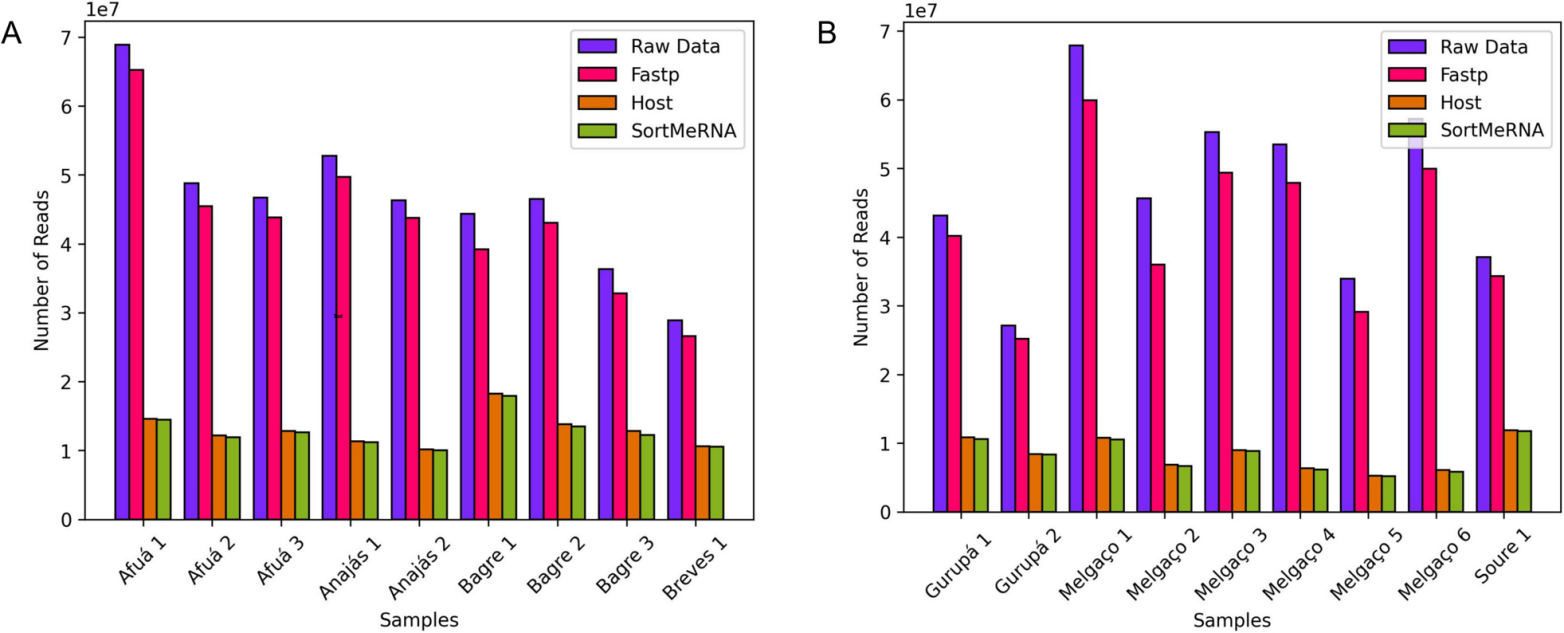
Raw reads counts and counts after software steps. **A** and **B** illustrates the amount of reads before and after the Fastp filtration, host genome mapping with Bowtie2 for removal, and removal of ribosomal RNA using SortMeRNA to facilitate the matching of reads with viral sequences. The Melgaço pools showed the lowest quantity of reads

**Fig. 2:**
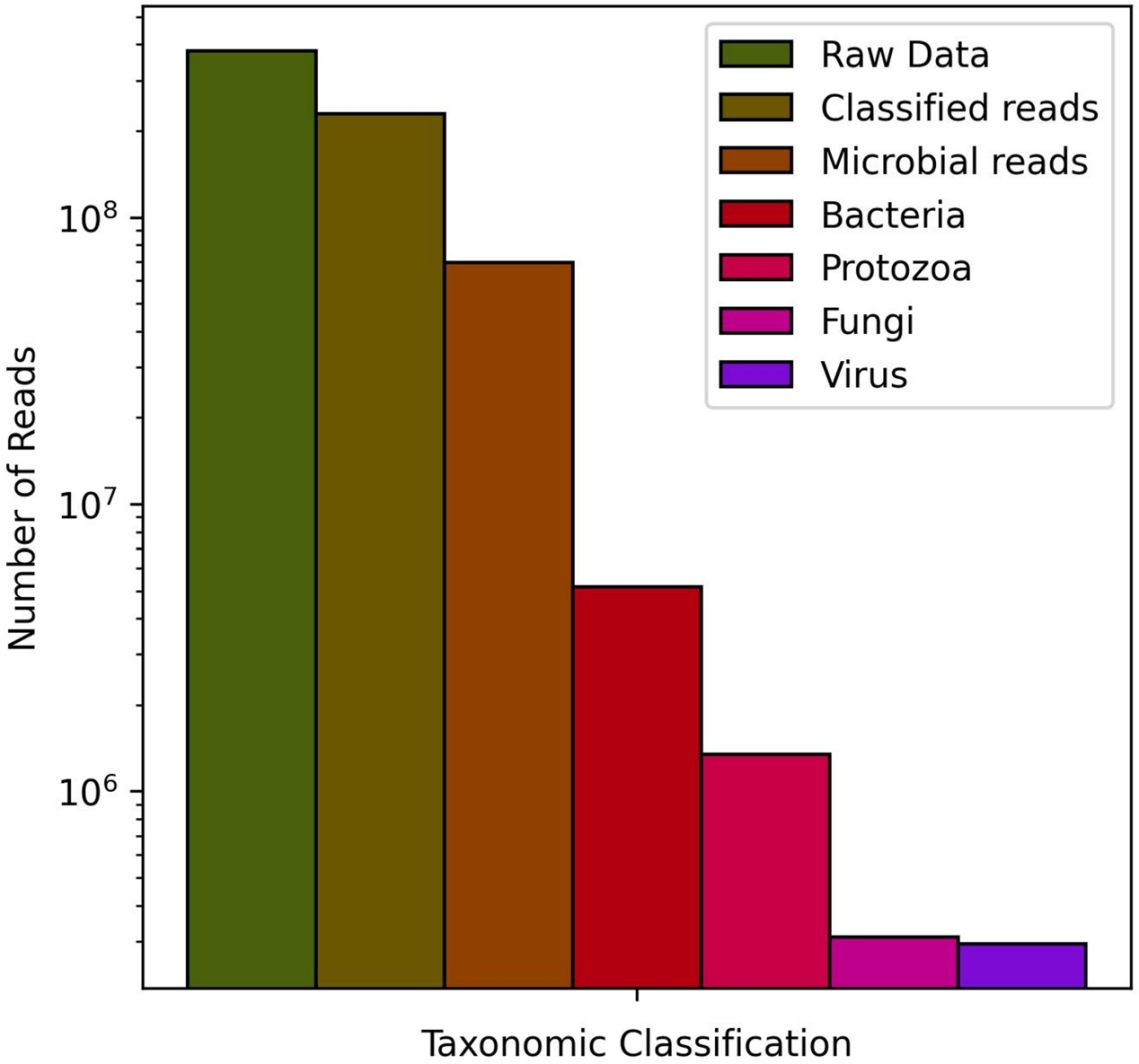
The quantity of raw reads and reads classified by taxonomic classification software. It is categorized into bacteria (red), protozoa (pink), fungi (dark pink), and virus (purple) taxa. Among these, the viral reads exhibited the lowest quantity

**Fig. 3:**
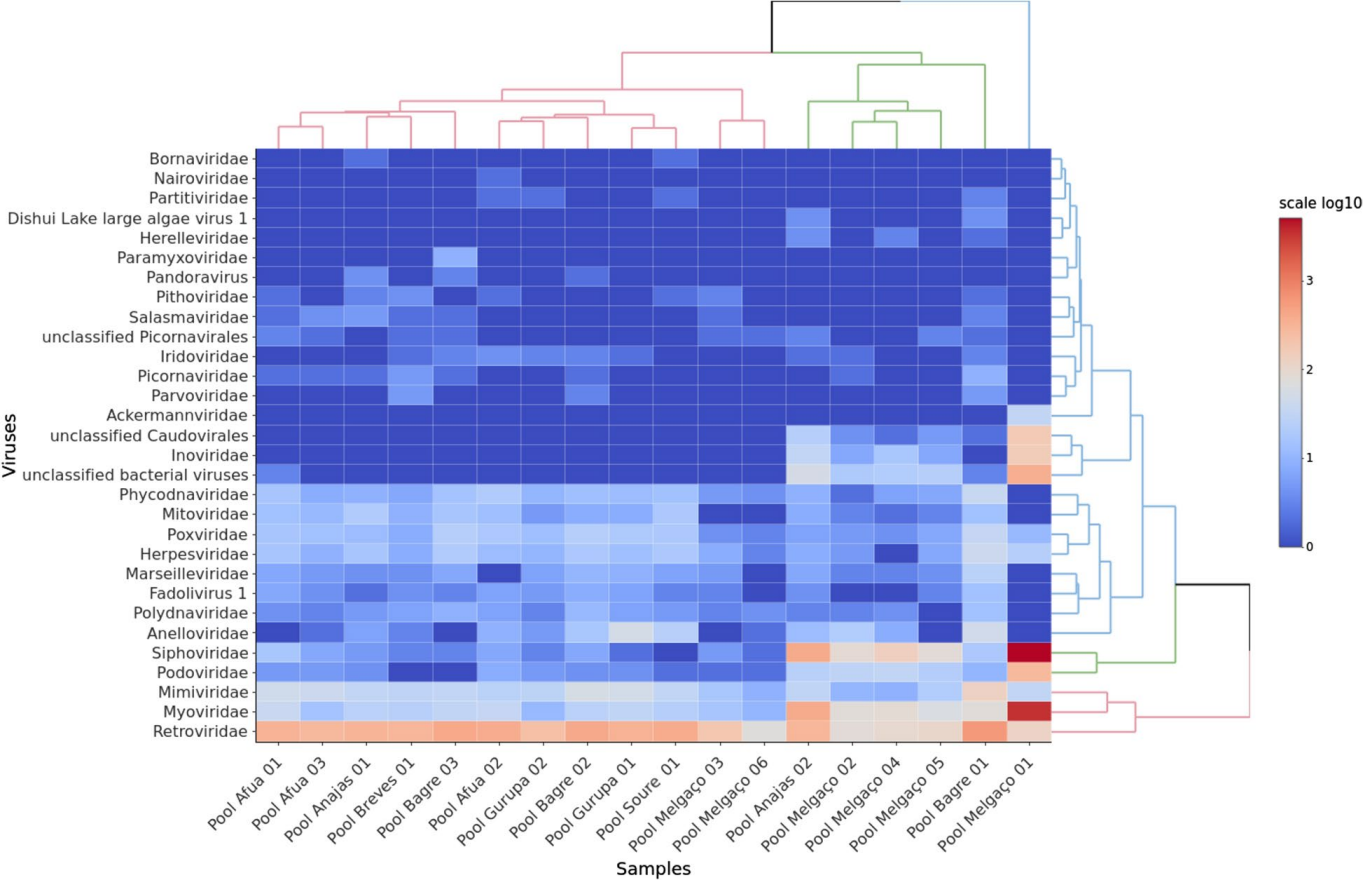
The heat map represents the distribution of reads for viral families. The figure displays the viral families that were detected and reads that could not be classified into families, such as *Caudovirales*. The quantity of reads is shown using a color gradient ranging from red, indicating the highest quantity, to blue for families with the lowest quantity. *Myoviridae, Podoviridae*, and *Siphoviridae*, which are among the eight phage families detected, as well as *Mimiviridae* that infects protists, had a significant number of reads. The Melgaço 1 sample exhibited the highest read counts for *Siphoviridae* and *Myoviridae*, as depicted by the red rectangles in the bottom right corner of the heatmap. On the other hand, RNA viruses were predominantly represented by the *Retroviridae* family across all samples

We obtained 840,125,868 raw reads sequences, and after the reduction steps, 188,423,522 reads were used for de novo assembly. Considering the relative amount, the pools from Melgaço had the greatest reduction (Fig. 1). The taxonomic classification programs estimated 69,252,530 microbial reads, with the lowest amount being for viruses, with 294,124 (Fig. 2). The alignment resulted in considerable viral diversity at different taxonomic levels when visualized in MEGAN. Reads corresponding to 24 families and other unclassified viruses can be observed in the heat map (Fig. 3). The number of contigs obtained by each assembler and the taxon classification were available in the Tables S1 to S4 (see Additional file 1).

### Overview of virome

Many reads and contigs matched phages. In total, eight phage families were detected: *Herelleviridae, Salasmaviridae, Achermamviridae, Inoviridae, Phycodnaviridae, Siphoviridae, Podoviridae, Myoviridae*, and unclassified *Caudovirales*, all with double-stranded DNA (dsDNA). All samples had the highest number of reads corresponding to *Myoviridae, Podoviridae*, and *Siphoviridae*, mainly in Melgaço 1 pool. This sample also showed the highest number of reads for *Mimiviridae*, which infects protists. Few families were related to infecting plants, fungi, and protozoa, such as *Mimiviridae, Partiviridae, Pithoviridae*, also with dsDNA genome, and *Mitoviridae* with single-stranded RNA (ssRNA).

Nine vertebrate viral families were identified, with dsDNA viruses predominating, related to *Iridoviridae*, *Poxviridae*, *Herpesviridae*, *Marseilleviridae*, and *Polydnavirida*. It was detected that only two families of single-stranded DNA (ssDNA) viruses exist, *Parvoviridae* and *Anelloviridae*. *Bornaviridae, Nairoviridae, Paramyxoviridae, Retroviridae*, and a group of unclassified *Picornavirales* were the single-stranded RNA (ssRNA) virus families identified. Some of them are able to infect invertebrates too.

Despite the significant number of reads, the marjority of families had very short contigs, which could represent low presence of these organisms or identity in the alignment. Therefore, we did not consider the species-level attributions reliable for most of them.

Even though the sequencing focused on RNA viruses, numerous families corresponding to DNA genomes were detected. This could be attributed to the imprecision of total RNA extraction kits, which were not entirely reliable or capable of detecting all transcripts. However, RNA viruses were strongly represented by *Retroviridae*, especially for the *Betaretrovirus* genus with 7,444 reads and 1,682 contigs and *Gammaretrovirus* with 22,796 reads and 6,592 contigs. Despite the abundance of *Gammaretrovirus*, the assemblers generated short contigs with an average length of 150 nt and low similarity to sequences in the database. On the other hand, *Betaretrovirus* obtained larger contigs with 95-99% similarity.

### Viral characterization and phylogenetic relationships of selected virus

During the inspection of the *Betaretrovirus* contigs, homologous sequences to the *Desmodus rotundus endogenous retrovirus* (DrERV) were discovered, with identity ranging from 95% to 99%. To obtain complete sequences of DrERV from all pools, the reference genome acquired in a previous study with *D.rotundus* from different locations in the Amazon (accession number MH648003) was used. De novo mapping was performed against the reads from each sample pool. The study sequences had an average length of 8,586 nt, which falls within the expected range for the *Betaretrovirus* genome length. The average GC content of the sequences was 49%.

**Fig. 4:**
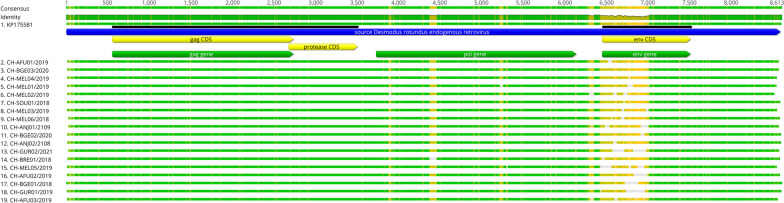
Alignment of the study sequences with DrERV genome (KP175581) from NCBI database. The reference sequence is represented by yellow bars, indicating the presence of Gag, Pro, Pol, and Env genes. The green bars represent the corresponding genes detected in the bat sequences from this study. Among these genes, the Env gene exhibited the highest amino acid divergence, observed in all sequences. Each sequence is identified by the location of origin and the year of collection

The Gag gene had an average length of 2,186 nt, Pro had 836 nt, Pol had 2,387 nt, and Env had 1,070 nt. The GC content of each coding region ranged from 45.7% to 52.11%. Among the sequences, Gag and Pro had intact Open Reading Frames (ORFs), while Pol had some stop codons and Env was the coding region that exhibited the most divergence between positions 7,463 to 8,279, which was an expected result since the reference sequences also displayed this variability (Fig. 4). Another alignment showing the gaps and diversity regions in the Env gene in more details (Additional file 2).

**Fig. 5:**
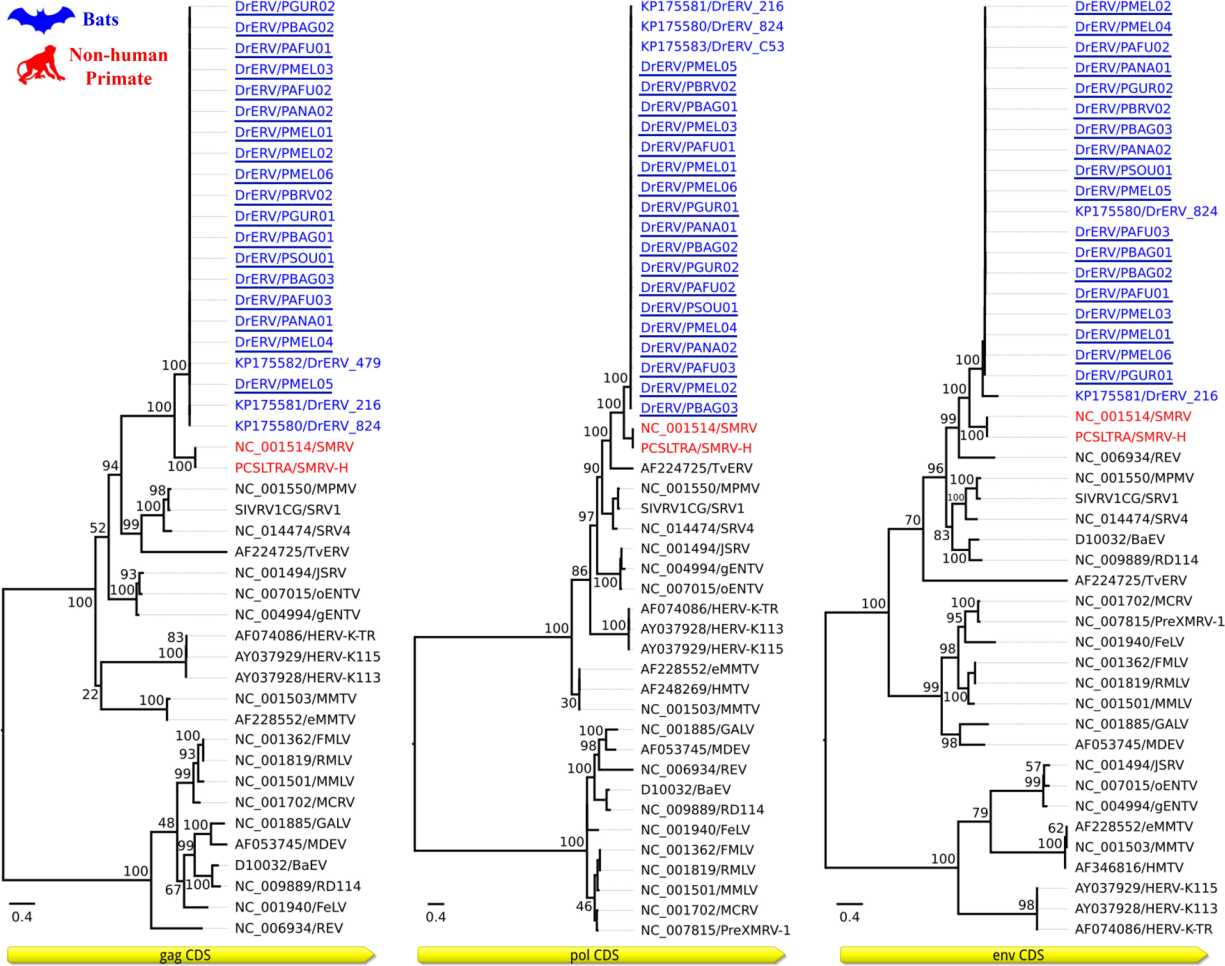
Phylogenetic inference performed based on the coding regions Gag, Pol, and Env. The trees show sequences of *Betaretroviruses* from bats and primates that were obtained from NCBI. The numbers at each main node in the tree correspond to bootstrap values in percentage (1000 replicates). The scale bar corresponds to the nucleotide divergence per site between sequences. The sequences from this study formed a monophyletic clade with other bat DrERV sequences from the database, as indicated by the underlined blue color. Meanwhile, the SMRV sequences clustered in a nearby branch, forming a paraphyletic group with non-human primate *Betaretroviruses* in all trees, highlighted in red, indicating a certain phylogenetic correlation

In the phylogenetic inference, 18 DrERV sequences were aligned with various *Betaretrovirus* strains accessible in the NCBI, with information available in Table S5 [see Additional file 1]. The Gag, Pol, and Env coding regions were used to generate three trees. The 18 sequences formed a monophyletic clade with other bat DrERV sequences, while the *Squirrel Monkey Retrovirus* (SMRV) sequences positioned themselves in a nearby branch, formed a paraphyletic group with primate *Betaretroviruses* (Fig. 5).

## Discussion

Bats serve as the primary natural host for a diverse range of mammalian viruses and play a crucial role in transmitting numerous emerging or re-emerge virus to humans and other animals in their natural habitats. Traditional virological methods identified many bat-borne viruses worldwide, which took multiple research laboratories and several decades to identify. However, with the introduction of metagenomic analysis utilizing NGS and high-throughput screening, the discovery of new bat viruses has greatly accelerated [2].

While feces, urine, oral swabs, and blood are commonly used for analysis, studies involving tissue samples are not as prevalent. The type of sample can affect the amount of information obtained, with fecal and intestinal samples typically producing more reads [16, 17].

The viruses infecting vertebrates stand out in metagenomic studies. Consistent with our analyses, families of DNA viruses, such as *Anellovirida* and *Parvoviridae*, were identified in fecal and saliva samples from *D. rotundus* in microhabitats of the Amazon region in French Guiana, with abundance varying across sampling locations. RNA viruses were less represented. This variation in the presence of DNA and RNA families is associated with the types of samples and the RNA depletion protocols using commercial kits [12].

A substantial number of reads and contigs in this study were linked to phages of *Myoviridae*, *Siphovirida*e, and *Podoviridae* families. It has been observed in other studies involving *D. rotundus*, but these DNA families are also frequently reported in non-hematophagous bats such as *Molossus*, *Myotis*, and *Rhinolophus* [12, 18, 19]. The prevalence of these families likely reflects a common state of phage infection in bat bacterial microecosystems across all studied regions and may indicate active replication within the hosts.

Identification of the RNA virome was the main focus, as viruses with simple genome can easily cross species barriers due to their high mutation rates. Thus, the occurrence of emerging viruses from bats demonstrates a high incidence of morbidity and mortality [11]. But the viral characterization could also be affected by rRNA depletion using laboratory kits [16]. Therefore, the removal of host rRNA and other organisms in the laboratory enabled the detection of more RNA taxa and improved sequencing depth for virus detection. This technique would be an option to ensure that more RNA viruses were detected in this study.

The lung is used in isolation in a few studies and is important because it is involved in the exchange of pathogens through the airborne route. Although, analyses of this organ suggesting their potential significance in detecting viruses associated with specific tissues, such in *Retroviridae* and *Nairoviridae* families [17, 20]. The present study identified 9 out of 24 viral families as being of vertebrate origin, mainly *Retroviridae*. This quantity is slightly lower than that reported in other metagenomic studies using bat tissues, it could be attributed to the choice of a single organ for analysis, while other studies have utilized multiple tissues to create the pools [17, 18].

The hematophagous diet of bats facilitates exposure to blood-borne viruses that can lead to an increase in the invasion of endogenous retroviral and non-retroviral elements in their genome. However, the diversity of endogenous retroviral elements is not exclusive to hematophagous animals. Several endogenized *Betaretroviruses* were detected in *Megachiroptera* and *Microchiroptera*, which have an insectivorous and frugivorous feeding habit. During the evolution of species, exogenous retroviruses infect germ cells and leave behind elements known as ERVs. The use of high-throughput sequencing techniques has rapidly increased the number of identified ERVs, with approximately 51 different bat species having been reported to carry ERVs [15]. In this study, *Gammaretrovirus* reads outnumbered *Betaretrovirus*, but all assembled contigs had low identity with corresponding sequences, preventing any inference in this genus.

The DrERV was first detected in samples of * D. rotundus* from Mexico and Germany, with sequences exhibiting intact ORFs for Gag and Pro genes, while Pol and Env exhibited several stop codons that resulted in truncated products [21]. The DrERV sequences from this study also presented stop codons in the Pol and Env genes, a characteristic of endogenous retroviruses, as most are unable to generate infectious particles. Moreover, both exhibited variability among the sequences. The Gag and Pro genes had a similar length to the reference sequence used in the mapping, while Pol and Env were slightly shorter with 2,186 and 1,070 nt, respectively, compared to other described sequence in brazilian vampire bats, with 2,418 and 1,770 nt [22].

*Carollia perspicillata*, also from the *Phyllostomidae* family, share a 75% ORF global nucleotide similarity with DrERV. However, such similarity did not occur with other *Phyllostomidae*, such as *Diphylla ecaudata* and *Artibeus jamaicensis*, suggesting that the insertion of DrERV occurred through independent infections after species divergence. Nonetheless, further investigations with a larger sample size are needed to represent the diversity of species found in this group. The DrERV sequences showed some similarity with the neotropical primate SMRV. In the first evidence of DrERV, the reads were mapped against the SMRV genome, and then phylogenetic inference was performed for the complete genome assembly [21]. At this study, the Gag, Pol and Env trees also formed a cluster with SMRV.

An analysis of thousands of ERVs in mammalian genomes demonstrates that bats play an important role as hosts of retroviruses and are involved in frequent events of cross-species transmission. Therefore, the presence of ERVs can aid in understanding evolutionary issues of the species that carry them. The discovery of endogenous or exogenous retroviruses in bats also raises the possibility of the existence of infectious retroviruses for humans hosted by these animals that are yet to be discovered [15].

The identification of *Adenovirus*, *Parvovirus* and *Paramyxovirus* is commonly described in studies with bats, some with brazilian bats [23–25]. In our analysis, even the presence of reads belonging to these families, along with the small contigs, did not aid in further analysis of the possible sequences carried by the *D. rotundus* in this areas.

Publications depicting viral diversity in *Vespertilionidae*, *Rhinolophidae*, and *Pteropodidae* have brought public health benefits by discovering the source of outbreaks. Bats from various places in Asia have shown bat-CoVs phylogenetically related to SARS-CoV-2, the causative agent of COVID-19 [11, 26]. Phyllostomid bats have interesting virome data, studies are focused on *D. rotundus*, as they are the main wildlife reservoirs of *Lyssavirus rabies* in the Americas. In the latest update of "The Database of Bat-associated Viruses”, 281 viral sequences are related to *D. rotundus*. Moreover, in the region search, 767 sequences are from different bat species in Brazil, a number much higher than in other South American countries [27].

The samples were collected from municipalities that can only be accessed by river transportation and are among the areas with the lowest Human Development Index (HDI) in Brazil, according to the United Nations Development Program. Melgaço has the lowest HDI, while others are associated with inappropriate management of solid waste, which causes damage to the environment and public health. This factor contributes to hypotheses about the virome characteristics harbored by circulating bats in the region since the contamination and transmission of viruses and other associated microorganisms may be favored in this scenario, as we can see from the readings referring to phages in this work [28, 29].

The traditional economy based on nature-extracted products necessitates entering forested areas, which puts the population at risk of attacks by hematophagous bats. Additionally, due to the low HDI and limited access to sanitation and quality housing despite the expansion of municipalities for extractive activities, locals are at an increased risk of contracting zoonotic diseases from wildlife. Recent studies have detected viruses belonging to the *Alphacoronavirus, Orthoparamyxovirus, Dependoparvovirus, Mastadenovirus,* and *Betaretrovirus* in Brazilian *D. rotundus*. However, it is crucial to emphasize that the majority of studies are conducted using samples from the state of São Paulo, and even though comprising half of the researches conducted in the country, they do not provide a comprehensive characterization of the virome carried by Brazilian bats [9, 23–25, 30, 31].

Despite the absence of families known to cause infections in mammals in this study, the identification of reads associated with other viral families is crucial for virological surveillance. Surveillance focused on wild animals in regions with environmental imbalances is necessary to comprehend the risk of contact between hosts of zoonotic viruses, domestic animals and humans [32]. This metagenomic study may initiate the understanding of the viral diversity carried by *D. rotundus* in the northern region of Brazil.

## Conclusion

These data are important for comparative analyses of bat virome obtained between lung and other types of samples. It was possible to correlate the presence of a common endogenous retrovirus in all tested samples, which may serve as a basis to aid in understanding the host genome. Phylogenetic analyses record that *Betaretroviruses*, such as DrERV, are present in neotropical bats and are a source of understanding the evolution of bats and other mammals, as well as may be related to other intrinsic factors of the species that still need to be studied.

## Methods

### Sites and sample collection

**Fig. 6:**
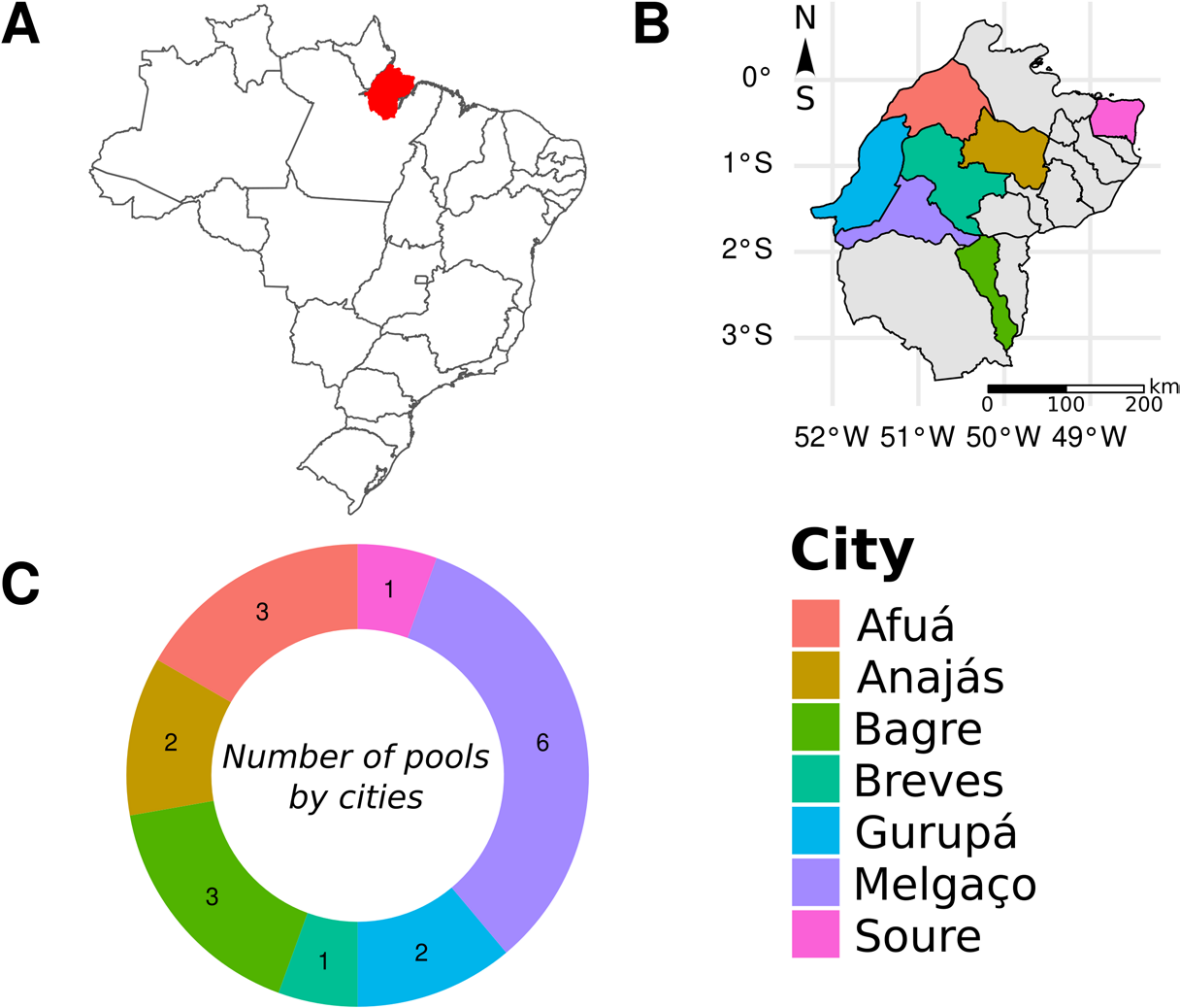
Localization and organization of the pools. **A** The figure displays the map of Brazil, and the red area is a part of Pará state. **B** The right-hand side illustrates an approximate map of the Marajó Island region, with colored areas indicating the municipalities where bats were collected for analysis. Although the Marajó Island region encompasses a total of 16 municipalities, only 7 were sampled for this study. **C** The pie chart illustrates the number of pools, each containing fragments from 3 to 5 bat lungs

Lung samples of 78 *D. rotundus* were collected between 2018-2021 and send to Evandro Chagas Institute (IEC) for routine surveillance and detection of *Lyssavirus rabies*. All these samples were negative for this virus and were grouped in 18 pools taking a fragment of lung organized by year and collection locate. Bats were captured in Melgaço, Afuá, Anajás, Breves, Bagre, Gurupá, and Soure, which are located in Marajó, the largest fluvial-maritime island in the world, situated in northern Brazil (Fig. 6). The utilization of bats was approved by the Animal Use Ethics Committee of IEC, certificate number 06/2022, and adhered to the prescribed research protocols in accordance with the ARRIVE guidelines [33].

### RNA preparation, cDNA synthesis and sequencing

Total RNA was extracted using TRIzol™ Plus RNA Purification Kit following owing manufacturer’s recommendations. RNA concentration was measured using Qubit® RNA HS Assay Kit in Qubit® 2.0 Flurometer (Invitrogen) and RNA integrity was assessed using the RNA Agilent RNA 6000 Pico Kit of the Bioanalyzer 2100 system (Agilent Technologies). For the synthesis of complementary DNA (cDNA), the SuperScript VILO MasterMix (Invitrogen) was used for the first strand, while the NEBNext mRNA Second Strand Synthesis Module (New England Biolabs) was used for the second strand. Then, cDNA was purified using AMPure beads. Sequencing libraries were generated using Nextera XT DNA Library Preparation Kit for Illumina®, following manufacturer’s recommendations. The library quantification was performed with the Qubit® dsDNA HS Assay kit, and the fragment sizes were analyzed using the High Sensitivity DNA Analysis Kit on the Bioanalyzer 2100 (Agilent Technologies). Following this, sequencing was performed on an Illumina® NextSeq 500 platform using the NextSeq 500/550 High Output v2.5 (300 cycles) kit and paired-end reads were generated.

### Bioinformatic analysis

The quality of the raw reads was evaluated using Fastp [34], which filtered out short reads of low quality and undetermined bases with a threshold of 20 and a length criterion of 60%. The reads were then mapped against the host genome (SM294091V2 Feb. 2018) and removed using Bowtie2 [35]. Ribosomal RNA was removed using SortMeRNA v.2.1 [36], and the quality of the resulting reads was re-evaluated using Fastp. The reads were aligned using DIAMOND [37] to compare with the viral non-redundant (NR) sequence database from the National Center for Biotechnology Information (NCBI) [38] and the taxonomic classification were evaluated using MEGAN v.6.23.2 [39]. The Kraken program was also used for taxonomic classification and Pavian was utilized for interactive data table, heatmaps and flow diagrams [40, 41]. De novo assembly was performed using SPAdes and MEGAHIT with k-mers size of 21, 33, 55, 77; and 21, 31, 41, 51, 61, 71, 81, 91 and 99 [42, 43]. The Quast program was used to generate the metrics of assemblers. The obtained sequences were realigned using DIAMOND and the contigs were inspected in MEGAN using the lowest common ancestor (LCA) assignment parameter with a maximum value of $10^{-10}$ to verify their classification. The resulting alignment was imported into GeneMarkS for gene prediction and InterProScan was used to determine the functional domains of the proteins [44, 45].

### Phylogenetic analysis

Reference genomes or nucleotide sequences of previously identified viruses from *Betaretrovirus* genus were downloaded from GenBank [38]. Alignments of nucleotide sequences with the study sequences were subjected to Mafft v.7 and manually inspected for corrections using the Geneious v.9.1.8 [46]. The aligned dataset was analyzed to identify the phylogenetic signal and determine the best nucleotide substitution model. Phylogenetic trees were constructed using Maximum Likelihood (ML) with IQ-TREE v.1.6.12 [47, 48]. Additionally, the bootstrap test was conducted with 1000 replicates and visualization was performed using FigTree v.1.4.4, employing the midpoint rooting methodology [49].

### Supplementary information

**Additional file 1.**

**Additional file 2.**

## Data Availability

The raw data of Brazilian bats have been submitted to the National Center for Biotechnology Information under BioProject PRJNA985242, BioSamples SAMN35848906 to SAMN35848923, SRA SRR25239926 to SRR25239943, and all sequences under accession numbers OR344913 to OR344930.
