# Supplementary material for: Virome analysis of Desmodus rotundus tissue samples from the Amazon region
Source: BMC Genomics. 2024 Jan 4;25:34. doi: 10.1186/s12864-023-09950-w (PMC10768307; doi:10.1186/s12864-023-09950-w)
Supplement: Supplementary file 2 — Additional file 2. [file 12864_2023_9950_MOESM2_ESM.pdf]

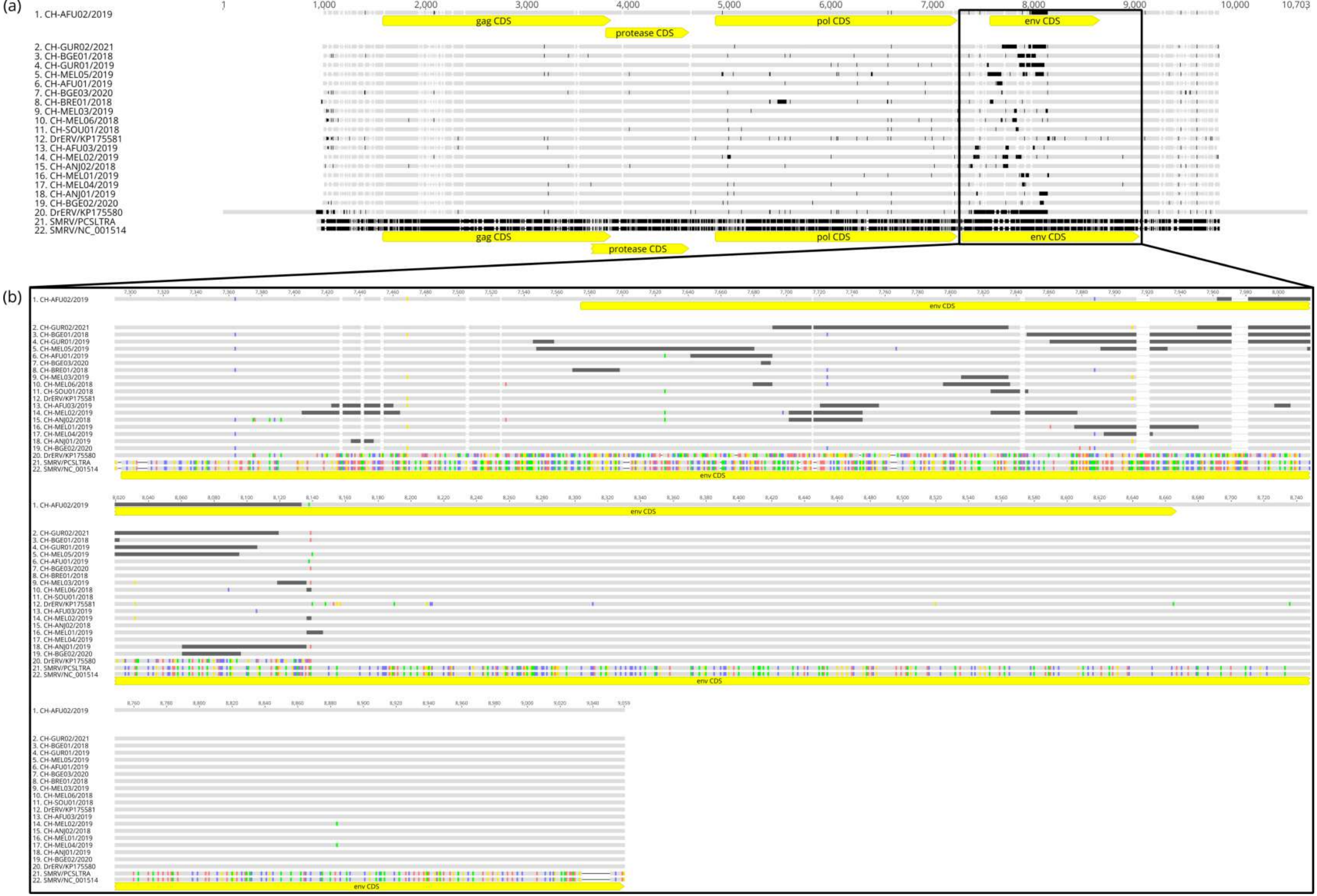


Fig. S1 **Aligment of sequences.** Alignment of the study sequences, and other DrERV and SMRV nucleotide sequences from the NCBI database. (a) The ORF is represented by yellow bars, indicating the presence of Gag, Pro, Pol, and Env genes. (b) Corresponding to the env gene, when light gray indicates identical sites, and dark gray represents regions of gaps; the color bar corresponds to the diversity of sites. Each sequence is identified by the location of origin and the year of collection.
